# Recent Trends in Food Quality and Authentication: The Role of Omics Technologies in Dairy and Meat Production

**DOI:** 10.3390/ijms26094405

**Published:** 2025-05-06

**Authors:** Ailín Martínez, Michel Abanto, Nathalia Baptista Días, Paula Olate, Isabela Pérez Nuñez, Rommy Díaz, Néstor Sepúlveda, Erwin A. Paz, John Quiñones

**Affiliations:** 1 Doctoral Program in Science Major in Applied Cellular and Molecular Biology, Universidad de La Frontera, Av. Francisco Salazar 01145, Temuco 4811-230, Chile; a.martinez26@ufromail.cl; 2 Meat Quality Innovation and Technology Centre (CTI-Carne), Universidad de La Frontera, Av. Francisco Salazar 01145, Temuco 4811-230, Chile; p.olate02@ufromail.cl (P.O.); i.perez04@ufromail.cl (I.P.N.); rommy.diaz@ufrontera.cl (R.D.); nestor.sepulveda@ufrontera.cl (N.S.); 3 Scientific and Technological Bioresource Nucleus BIOREN-UFRO, Universidad de La Frontera, Av. Francisco Salazar 01145, Temuco 4811-230, Chile; michel.abanto@ufrontera.cl (M.A.); nathalia.dias@ufrontera.cl (N.B.D.); 4 Doctoral Program in Agrifood and Environment Sciences, Universidad de La Frontera, Temuco 4811-230, Chile; 5 Faculty of Agricultural and Environmental Sciences, Universidad de La Frontera, Av. Francisco Salazar 01145, Temuco 4811-230, Chile; 6 UWA Institute of Agriculture, The University of Western Australia, Perth 6009, Australia; erwin.pazmunoz@uwa.edu.au

**Keywords:** omics approach, functional foods, milk, meat, quality, authentication, fraud, adulteration

## Abstract

The global demand for animal protein presents significant challenges in the production of nutritionally rich foods, such as milk and meat. Traditionally, the quality of these products is assessed using physicochemical, microbiological, and sensory methods. Although effective, these techniques are constrained by time limiting their widespread application. Furthermore, growing concerns regarding sustainability, animal welfare, and transparency have driven the development of technologies to enhance the rapid and precise assessment of food quality. In this context, omics technologies have transformed the characterization of animal-origin food by providing in-depth molecular understanding of their composition and quality. These tools enable the identification of biomarkers, adulteration detection, optimization of nutritional profiles, and enhancement of authentication and traceability, facilitating the development of functional foods. Despite their potential, several barriers persist, including high implementation cost, the need for specialized infrastructure, and the complexity of integrating multi-omics data. The main aim of this review was to provide information on advances in the application of omics technologies in dairy and meat production systems and studies that use them in food quality, authentication, and sustainability. It also outlines opportunities in areas such as fraud prevention and functional product development to support the transition to safer, healthier, and more transparent food systems.

## 1. Introduction

Currently, the growing global demand for animal protein poses significant challenges to produce foods such as milk and meat, which are recognized for their high nutritional value as sources of high biological value proteins, essential fatty acids, vitamins, and minerals [1,2]. Cattle represent one of the main sources of animal production worldwide, with wide genetic diversity, as there are more than 1000 breeds distributed globally. In 2022, the world cattle population amounted to 1.55 billion head [3]. In this context, accurate analysis of the quality and safety of these products has become indispensable to ensure their authenticity, nutritional value, quality, and safety for human consumption.

Food quality assessment has been based on physicochemical, microbiological, and sensory methods, which, although effective, have limitations in terms of cost and time [4]. In addition, a growing interest in sustainability, animal welfare, and transparency in production processes has driven the development of advanced technologies for faster and more accurate assessments [5]. These innovations not only improve quality control but also facilitate the monitoring of nutritional and organoleptic aspects in line with consumer demand [6].

In this framework, omics technologies enable the comprehensive characterization of molecular systems, including the analysis of complete genomes (genomics, complete sets of RNA (transcriptomic), proteins (proteomic), and metabolites (metabolomic). These technologies have revolutionized the characterization of animal products by providing a comprehensive understanding of their composition and quality [7]. These advanced tools facilitate the examination of genetic, protein, and metabolic aspects at the molecular level, facilitate the detection of bioactive compounds, identify adulteration, and improve traceability, thus responding to consumer expectations for safer, more nutritious, and high-quality foods [8,9,10].

However, their implementation faces several challenges because of the high costs of sequencing and analysis tools as well as the need for specialized equipment and trained personnel, especially in developing regions [11,12,13]. Another key challenge is the integration and analysis of multi-omics data, which are often heterogeneous and high-dimensional. This requires the use of advanced computational techniques such as machines and deep learning, which require expertise that is not yet accessible in many contexts [14].

Despite these limitations, technological advances and collaboration among researchers have increased the accessibility and efficiency of omics. Genomics, for example, plays a key role in breeding programs and food product authentication [15,16], whereas proteomics and metabolomics offer valuable tools for optimizing the nutritional quality, sensory properties, and safety of milk and meat [10,17]. Likewise, lipidomics enables the precise control of fatty acid profiles, promoting the production of functional foods [18].

The integration of these disciplines into multi-omics approaches provides a holistic perspective for assessing and ensuring the quality and safety of dairy and meat products, favoring the optimization of their preservation and traceability [19]. These advanced tools not only deepen the understanding of biochemical pathways associated with food production but also facilitate the development of functional products by improving their nutritional profiles and ensuring high-quality and safety standards. Furthermore, omics technologies facilitate the identification of bioactive compounds with beneficial properties and optimization of manufacturing processes, resulting in higher quality products with significant added value. Its main advantages are its speed and accuracy in the analysis of food components compared with traditional methods. In addition, the integration of complementary omics disciplines enables a comprehensive assessment of food quality and safety through a more efficient approach [20]. Its main advantages are its speed and accuracy in the analysis of food components compared to traditional methods. In addition, the integration of complementary omics disciplines enables a comprehensive assessment of food quality and safety through a more efficient and cost-effective approach [10,21,22].

This approach responds to consumer demands and regulatory requirements and promotes safer, healthier, and more sustainable food. In this context, this paper aims to review the literature on the application of omics technologies in milk and meat production and the most recent studies exploring their contribution to improving the quality, safety, and authenticity of these products.

## 2. Overview of Omics Technologies

### 2.1. Current Development of Omics Technologies

Omics technology refers to a set of advanced analytical methods used to study the complete profile of biological molecules [23]. Approaches based on these technologies in the field of food production are known as food omics and are used for the in-depth characterization and efficient management of various food products. Food omics integrates food and nutritional studies through the application of omics technologies to improve the quality and ensure the safety of fresh food and processed products, thereby promoting the health and well-being of consumers [21]. By combining data from these diverse analytical platforms, omics enables a holistic understanding of how dietary ingredients influence cellular processes, metabolic pathways, and functional mechanisms within the organism, which not only facilitates the identification of biomarkers and elucidation of bioactive compound effects but also enhances food quality, safety, authenticity, and traceability [24].

These technologies allow for an expanded knowledge of the biological pathways and mechanisms involved in the development and determination of intrinsic food quality traits [22,25,26]. This facilitates a deeper understanding of the variations in quality and origin of defects, adulteration, or contamination that may compromise its attributes. Tools such as those that are genomic, transcriptomic, proteomic, peptidomic, metabolomic, and lipidomic provide comprehensive high-throughput data on foods’ composition, safety, and nutritional value [27]. These data, combined with advanced statistical analyses and bioinformatic methods, provide a comprehensive and detailed view of products and strengthen their traceability and quality control [21,23].

In the milk and meat production context, the sector faces multiple challenges related to food security and environmental sustainability, including population growth, scarcity of natural resources, climate change, and the need to optimize land use and the carbon and nitrogen cycles [28]. To address these challenges, the adoption of innovative technologies such as omics offers fundamental tools to deepen the understanding of biological systems involved in animal food production. Implementing these approaches supports the development of more sustainable and efficient production practices, improves productivity, and helps mitigate environmental impacts.

### 2.2. Key Omics Disciplines and Analytical Approaches

Genomics focuses on studying the structure, function, editing, and evolution of genomes through a detailed understanding of DNA sequences [29,30]. The focus is on analyzing the heterogeneity of coding genes and exploring their sequence and expression. Beyond identifying specific genes, this discipline seeks to understand interactions within complex biological systems. Genomics provides essential information on the molecular basis of diverse biological processes and has been central to recent advances in medicine, biotechnology, food science, food safety, and the agri-food sector [31]. The tools used in genomic studies include the Polymerase Chain Reaction (PCR) for amplification of specific regions, multilocus variable number tandem repeat analysis (MLVA), pulsed field gel electrophoresis (PFGE), and metagenomic assays such as 16S rRNA amplicon sequencing [32].

Proteomic analyses are based on two main approaches: top-down and bottom-up. A top-down approach was used to characterize the PTMs present in intact proteins. This method relies on the initial separation of proteins by liquid chromatography (LC) or two-dimensional gel electrophoresis, followed by their detection by MS. Intact proteins are ionized, fragmented in a collision cell, and subsequently analyzed by tandem mass spectrometry (MS/MS). In contrast, the bottom-up approach is characterized by the enzymatic digestion of proteins into peptides before MS analysis, enabling peptide/protein separation and identification using high-resolution mass analyzers such as Orbitrap^®^ and quadrupole time-of-flight (QTOF) instruments [33,34]. The development of advanced platforms, such as the Orbitrap Eclipse Tribrid MS, which integrates a high-performance quadrupole mass filter, dual-pressure linear ion trap, and Orbitrap mass analyzers, has significantly enhanced analytical capacity and flexibility [35]. Additionally, recent innovations in ion mobility mass spectrometry (IMMS), including technologies such as timsTOF, traveling wave IMS (T-wave IMS) on the SYNAPT G2-Si system, and field asymmetric IMS (FA-IMS) on Orbitrap platforms, have marked important advances in the field [36,37,38]. These emerging tools have contributed to greater resolution, accuracy, and depth in proteomic analysis.

Metabolomics focuses on analyzing changes in large sets of metabolites present in biological samples, providing a comprehensive view of metabolic processes and reflecting the physicochemical properties of the systems studied. Its main objective is to identify and quantify small molecules (usually <1000 Da) in biological systems [23]. For this purpose, two fundamental approaches were used: targeted and nontargeted metabolomics. The selection of an appropriate methodology for a metabolomic study depends on factors such as high analytical sensitivity, short chromatographic run times, direct infusion techniques, scanning speed, and mass resolution. The main technologies include LC-HRMS, GC-HRMS, CE-HRMS, UHPLC-Q-Orbitrap-MS, UHPLC-Q-ToF-MS, direct infusion mass spectrometry (DIMS), and nuclear magnetic resonance (NMR). Additionally, ultra-HR-MS approaches based on flow injections, such as Fourier transform ion cyclotron mass spectrometry (FT-ICR-MS), have been used to map the food metabolome. The most common interfaces in LC-coupled HR-MS-based metabolomics employ electrospray ionization (ESI) or heated electrospray ionization (HESI) sources, which generally operate in the positive mode [34].

Lipidomics, which emerged in 2003 as a subcategory of metabolomics [39], has established itself as a tool for characterizing lipids and other lipophilic compounds in cell membranes [40]. This discipline is classified into non-targeted and targeted lipidomics [41]. Untargeted lipidomics is used to analyze global changes in the entire lipidome of a system, providing a holistic perspective. Targeted lipidomics focuses on the characterization of specific lipids to address complex lipid-related biological problems. Its main applications include the characterization of lipids and their metabolites in biological systems, lipid changes associated with lipid function and metabolic regulation, and the search for lipid biomarkers relevant to lipid metabolism pathways and networks [42,43,44,45].

The data acquisition process in lipidomics typically involves the separation of lipid species through ionization or chromatographic techniques, followed by their identification and quantification. Lipid research has significantly advanced with the incorporation of cutting-edge analytical technologies such as spectroscopy and chromatography [46]. Among these, liquid chromatography coupled with mass spectrometry (LC-MS) is particularly notable for its high sensitivity and specificity, enabling the detailed identification and characterization of lipid profiles in meat products. This technique provides molecular ‘fingerprints’ that support quality control, traceability, authentication, and the differentiation of animal species based on their lipidomic signatures [47]. The most widely used tools in MS-based lipidomics include direct infusion mass spectrometry (DI-MS), chromatography coupled with MS, ion mobility mass spectrometry (IM-MS), and mass spectrometry imaging (MSI) techniques [48]. Additionally, nuclear magnetic resonance (NMR) spectroscopy serves as a complementary technique, providing structural information and supporting comprehensive lipid characterization.

## 3. Application of Omics Technologies in Dairy and Meat Production

### 3.1. Integrative Role of Omics in Food Quality, Nutritional Profiling, and Product Authenticity

#### 3.1.1. Genomics

As one of the core omics technologies, genomics provides advanced tools for the analysis of genetic profiles in livestock, thereby enhancing traceability, authenticity, and production efficiency in the agricultural sector. It plays a fundamental role in animal breeding programs by enabling the identification of genes associated with desirable traits such as disease resistance, productive efficiency, and the quality of derived products like milk and meat. Moreover, genomics contributes to determining the geographical origin of dairy and meat products, reinforcing their authenticity and increasing their added value [14,15,49,50,51,52,53,54,55].

This technology also facilitates the detection of specific genes linked to traits that directly impact the safety and nutritional quality of animal-derived foods [14]. Genomics is particularly effective for identifying genetic markers, genomic regions, and candidate genes associated with economically important traits, as well as for improving the accuracy of genetic value estimations in breeding populations [56,57]. Collectively, these applications of genomics contribute not only to the optimization of production systems but also to the assurance of food quality, safety, and traceability (Table 1).

In the field of dairy product authentication, genomics enables the identification of biomarkers based on animal DNA or breed-specific genetic signatures [58]. Genetic markers, such as single-nucleotide polymorphisms (SNPs) and microsatellites, are key tools for different species, such as cows, goats, and sheep. In addition to determining the origin of milk, these markers can provide information on the geographical origin and breed of animals [59]. The main genomic methods used for the identification of milk and milk products include PCR, real-time PCR, multiplex PCR, PCR with capillary electrophoresis (PCR-CE), and biosensors. These techniques have applications in the characterization of dairy products [60] and the detection of adulteration. However, despite these advantages, PCR has several limitations. The presence of high levels of fat and protein in samples can inhibit this reaction, making it difficult to detect non-DNA adulterants, which restricts their application in certain authentication methods [61].

In contrast, high-throughput DNA sequencing technologies have revolutionized the identification of genetic markers, enabling rapid and accurate authentication of milk and dairy products [62]. These techniques facilitate the detection of biomarkers associated with specific adulterants, such as the addition of water to milk, which can dilute the genetic material, alter its genomic composition, and aid in the identification of fraudulent practices [63]. Similarly, genetic markers for common adulterants like sugar, salt, and non-dairy proteins can be used to detect their presence in dairy products, ensuring consumer protection against fraudulent labeling [64]. However, these technologies have certain limitations, particularly the need for high-quality DNA to ensure effective amplification. Several next-generation sequencing (NGS) platforms are available for high-throughput sequencing, with Illumina being the most widely used [65]. Moreover, several open-source software tools for NGS data analysis, such as the Genome Analysis Toolkit (GATK) and SAMtools, are commonly employed [66]. The snpTree web server has been developed to streamline automated single-nucleotide polymorphism (SNP) analysis and phylogenetic tree construction, integrating tools like SAMtools and MUMmer to enhance accessibility for researchers in life sciences [67]. Despite its promising potential for food authentication, the application of NGS to dairy products remains limited [68].

Authentication and traceability have become essential pillars for ensuring quality, food safety, and consumer confidence in meat production and marketing. Over the past two decades, genomic selection (GS) has gained widespread acceptance in animal breeding programs worldwide due to its ability to increase selection accuracy, reduce the need for phenotyping, shorten breeding cycles, and maximize genetic gain [69]. Genome-wide association studies (GWAS) have provided invaluable insights into the genetic architecture of beef-related traits, linking specific phenotypic traits with genomic data and enabling the detection of molecular signatures for animal selection and breeding [70]. This integration of genomic tools has optimized decision-making in breeding programs, promoting more efficient and sustainable breeding strategies in response to the increasing demand for high-quality responsibly produced beef [71].

A recent genome-wide association study (GWAS) in Nellore cattle employed the WssGBLUP method to identify 10 key genomic regions. These regions explained 8.13–21.41% of the additive genetic variance in traits linked to carcass and meat quality. The analysis highlighted 715 candidate genes associated with traits such as loin eye area, backfat thickness, carcass weight, cutting strength, marbling, and intramuscular fat content. Genes such as CAST, PLAG1, XKR4, AQP3/AQP7, and MYLK2 are involved in essential physiological processes linked to growth, meat quality, and lipid metabolism and are promising candidates for breeding programs [72]. Another study conducted in the population of Holstein cows in the lower Yangtze River region analyzed milk yield, fat percentage and yield, and protein percentage and yield, showing heritability levels ranging from 0.22 to 0.31 for milk yield and 0.06 to 0.15 for fat percentage. Genome-wide association analyses identified SNPs responsible for a significant proportion of the phenotypic variation in these traits, with values ranging from 5.44% for milk yield to 12.39% for fat percentage. In addition, the enrichment of metabolic pathways (KEGG) and Gene Ontology terms related to the detected SNPs highlights the complexity of the genetic networks controlling these traits. Among the candidate genes identified, CAMK2G, WNT3A, and DGAT1 stand out for their potential impact on milk production and quality [73].

Genomics has also been used to identify genes associated with disease resistance and milk quality in dairy cattle. Several studies have explored different approaches to improving her health and productivity. Ali et al. [74] investigated polymorphisms in the bovine transferrin gene in Holstein Friesian cows, discovering genetic markers that may be useful for early detection of mastitis resistance or susceptibility. These findings have important implications for the structure and function of the transferrin protein, which is associated with the animal’s immune response. Rahayu et al. [75] analyzed single-nucleotide polymorphisms (SNPs) in the B4GALT1 gene in crossbred cattle from Kerala, India. Their research aimed to design specific breeding strategies to improve cattle genetics in the region by adapting them to local conditions and production needs.

Moreover, genetic selection and improvements have resulted in genetically superior disease-free animals with optimized production and efficiency for various livestock species [76,77]. Significant advances have been made in the field of molecular genetics, including the identification of multiple genes and gene combinations that are directly associated with animal performance and production efficiency [77,78]. In addition, numerous quantitative trait loci (QTL) responsible for the variability in various productive and reproductive traits have been identified and are key to breeding decisions [79]. In addition, several genetic markers have proven useful for marker-assisted selection (MAS) in breeding programs [80,81].

An important advancement in genomics is the detection of adulterants in food products. A recent study introduced a DNA barcode analysis method to identify the adulteration of vegetable oils in dairy products. This method uses a universal plant barcode as the amplification target, enabling the detection of undeclared plant material in dairy samples [82]. Two PCR-capillary electrophoresis (PCR-CE) assays based on the trnL intron of the plastid and its inner loop P6 facilitated the identification of maize, soybean, rapeseed, and sunflower oils in clarified butter, milk, and yogurt. The P6 analysis exhibited higher sensitivity, providing accurate species identification involved in adulteration.

Meat authentication is an important aspect for protecting consumers from illegal or unwanted ingredients. Recent studies have addressed the authenticity, origin, and detection of meat adulteration using genomic methods. Cai et al. [83] developed a simple and reliable single-tube mitochondrial DNA-based 7-fold PCR assay that simultaneously identified seven species: pork, beef, sheep, chicken, turkey, goose, and duck. This method was validated in terms of sensitivity, specificity, robustness, and low cost, demonstrating its potential for wide application in the detection of meat origins in foods suspected of adulteration. On the other hand, Dobrovolny et al. [84] conducted a collaborative study involving 15 laboratories. This study focused on harmonizing an analytical method based on DNA metabarcoding to detect the adulteration of poultry and mammalian meat. In the trial, each team analyzed 16 anonymized labelled samples, including six mixtures of DNA extracts, a DNA extract from a model sausage, and a maize extract used as a negative control. The evaluation parameters confirmed the reliability of DNA metabarcoding as a tool for authenticating meat species in routine analysis.

**Table 1:** Genomics as a strategic tool in dairy and meat production systems.

Food Matriz	Techniques/Methods	Results	Application	References
Milk and Dairy Products	Real-time PCR targeting mitochondrial 12S rRNA and cytB genes	High sensitivity and specificity for detecting species such as *Bos taurus*, *Ovis aries*, *Bubalus bubalis*, and *Capra hircus*. Detection limit <1%	Origin Identification	[85]
	Specific TaqMan probes for the detection of cow and mare DNA	Accurate and reproducible detection in koumiss and yogurt	Origin Identification	[86]
	Weighted single-step GWAS using genomic breeding values	Identification of 141 novel genes related to milk production and 5 associated with Somatic Cell Score	Quality Identification	[87]
	PCR-RFLP targeting κ-casein gene (CSN3)	Differentiation between cow, goat, and sheep milk	Authenticity Identification	[88]
	Conventional and real-time PCR for milk powder using 12S rRNA gene	Verification of mitochondrial DNA integrity in powdered milk	Adulteration Detection	[62]
Meat	CNV and CNVR detection using Bovine HD SNP array	Identification of 112,198 CNVs and 10,102 CNVRs, including regions related to backfat color and thickness	Composition Analysis	[89]
	GWAS and pathway-based analysis using GeneSeek Genomic Profiler Bovine LD array	37 significant SNPs associated with 12 traits in Piedmontese cattle	Quality Identification	[90]
	Genome-wide association studies (GWAS) on carcass traits	Identification of SNPs and genes linked to growth, muscle development, and meat quality	Quality Identification	[81]
	Multiplex PCR for meat authentication (7 species)	High reproducibility even in heat-processed meat; low detection limits	Authenticity Identification	[91]
	Two-tube hexaplex PCR for 12 meat species	Molecular identification of up to 12 species in adulterated meat mixtures	Adulteration Detection	[92]

Selection Criteria: Most recent scientific articles.

#### 3.1.2. Proteomics

Proteomics, one of the most advanced omics technologies, enables a detailed analysis of functional and bioactive proteins, providing critical information on the nutritional value, functional properties, and sensory quality of meat and dairy products [16,17,18,19,20,21,22,23,24,25,26,27,28,29]. The protein profile of milk is a key indicator of its nutritional and functional value, as many proteins, including caseins and lactoglobulins, possess antimicrobial and immunomodulatory properties that are beneficial to human health [93]. In the meat industry, proteomics offers tools for evaluating meat quality in terms of texture, flavor, and protein content, which are essential for satisfying consumer demand for high-quality products [94]. Moreover, this technology plays a crucial role in the detection of adulteration in meat and dairy products, reinforcing consumer confidence and mitigating public health risks associated with food fraud [8,9,10,11,12,13,14,15,16,17,18,19,20,21,22,23,24,25,26,27,28,29]. Additionally, proteomics has proven effective in identifying key allergens, such as lactoglobulins and caseins in dairy products, and tropomyosin in meat, contributing to enhanced food risk management and consumer safety [95,96] (Table 2).

A comprehensive proteomic analysis of small extracellular vesicles (EVs) present in milk from cows during the late lactation stage was performed using liquid chromatography coupled with mass spectrometry (LC-MS/MS). This study identified 2225 proteins, 429 of which were novel findings compared to those of previous studies. Known for their ability to transport proteins, nucleic acids, and lipids, EVs play a key role in cell communication and are involved in crucial biological processes such as immunity and mammary gland physiology. Bioinformatics analysis revealed that the identified proteins were associated with metabolic, immunological, and cellular regulatory functions. The changes detected in the protein profile of EVs reflect specific physiological adaptations during the preparation for the dry period and the subsequent lactation phase [97].

Another recent study analyzed the proteomic profile of whey from Jersey crossbred cows in India using high-resolution mass spectrometry and chromosome mapping techniques. More than 29 low-abundance proteins were identified, highlighting the molecules associated with metabolic processes, immune regulation, and defense responses. Bioinformatics analysis revealed that chromosomes 5 and 9 contributed significantly to protein expression, showing a high number of unique peptides. Relevant proteins, such as ubiquitin, desmoglein, annexin, and heat shock proteins (HSP-70), which are linked to thermotolerance and regulation of the immune system, were also identified [98].

The proteomics study by Zhu et al. [99] used proteomics technology to analyze and quantify the differences in protein composition of sheep, goat, and cow milk, identifying a total of 770 proteins. A significant variation in the abundance of 74 proteins was observed between the species. Among them, specific proteins, such as CSN3 and LALBA in goat milk, XDH in cow milk, and CTSB and BPIFB1 in sheep milk, were identified as biomarkers. Moreover, bioinformatics analysis associated these proteins with biological processes, including thyroid hormone synthesis, lipid metabolism, and innate immune response. On the other hand, Verma et al. [100] used nanoscale liquid chromatography-tandem mass spectrometry (nLC-MS/MS) for the identification of 1307 functional proteins comprising casein and other low-abundance proteins. The authors reported that many of these proteins are involved in binding, catalysis, and structural activities. Pourjoula et al. [101] carried out proteome and peptidome characterization of Kashz, a traditional Iranian dairy product made from sour milk. They used advanced technologies such as SDS-PAGE and high-performance liquid chromatography (HPLC) separation techniques coupled with mass spectrometry (MS). The results showed that the protein profile of Kashz was like that of yoghurt. Furthermore, in vitro hydrolysis analysis with plasmin revealed that Kashz is a typical Iranian dairy product, and its formation is due to an acidic coagulation process in which caseins are not involved.

Fang et al. [102] observed that the relative concentrations of phosphorylated αs-casein in cow’s milk vary as a function of intrinsic factors, such as parity and genetic variation, as well as extrinsic factors related to the stage of lactation. Using liquid chromatography coupled with electrospray ionization mass spectrometry (LC-ESI MS), these variables were shown to contribute significantly to phenotypic variation in individual phosphorylation isoforms and the overall degree of phosphorylation of αS-caseins. Post-translational modifications, such as phosphorylation, acetylation, glycosylation, disulfide bond formation, lipid conjugation, and proteolytic cleavage, play crucial roles in the functions of milk proteins. These modifications are essential for micellar stability, which is a key aspect of cheese manufacturing [103]. These differences in the levels of phosphorylation have a notable impact on the industrial properties of milk, highlighting their relevance in processes such as coagulation [29].

Picard and Gagaoua [104] conducted a proteomic study aimed at identifying protein biomarkers associated with beef tenderness. This work consisted of a comprehensive review of 12 experiments using the technique of two-dimensional electrophoresis (2-DE) combined with mass spectrometry to compare groups of beef with different levels of tenderness, specifically in the *Longissimus thoracis* and *Semitendinosus muscles* of different cattle breeds. The results of these studies revealed a total of 61 proteins with significant (*p* < 0.05) differential abundances between tender and tough meat groups. Of these, the *Longissimus thoracis* muscle showed a higher number of discriminative proteins (50) than the semitendinosus (28). According to Gene Ontology annotations, proteins involved in muscle structure, contraction, protection against oxidative stress, energy metabolism, and proteasome subunits are particularly involved in determining the tenderness of the Longissimus thoracis muscle. Among the biomarkers identified, proteins such as HSPB1 and muscle- and sex-specific biomarkers stand out, with parvalbumin being a particularly robust marker in the semitendinosus muscle. This integrated proteomic analysis not only provided a deeper understanding of the biological processes influencing meat tenderness but also proposed a comprehensive list of candidate biomarkers for further investigation.

Gagaoua et al. [105] reviewed how proteomics has advanced our understanding of the complex biological systems that affect beef color. This study reviews 13 independent investigations on muscle proteomics, creating a database that includes 79 proteins significantly correlated with meat color characteristics. Six biological pathways that influence meat color, including energy metabolism and oxidative stress, were identified. Prominent biomarkers include proteins such as β-enolase (ENO3) and Superoxide Dismutase (SOD1), which have consistently been reported in multiple studies.

Post-mortem muscle protein changes and their relationship to meat quality have also been investigated in steers and heifers using two-dimensional electrophoresis mass spectrometry in the Longissimus thoracis muscle. The results showed significant differences in carcass characteristics and chemical composition between the two groups, with heifers having greater subcutaneous fat thickness (46.8% more) and higher intramuscular fat content (63.6% more) than steers. Proteomic analysis identified differentially expressed proteins associated with energy metabolism, nutrient transport, and response to oxidative stress in heifers, suggesting a higher oxidative capacity and lower glycolytic activity compared to steers. In contrast, steers showed a higher abundance of proteins related to muscle contraction and ATP metabolism, indicating a higher glycogenolytic activity [106].

Zhu et al. [107] used proteomic techniques to identify biomarkers associated with the sensory quality of beef, specifically tenderness, juiciness, and chewiness. To this end, plasma samples and biopsies of the *Longissimus thoracis* and *Longissimus lumbrum* muscles of young Limousin bulls, classified into high- and low-quality groups according to their sensory attributes, were analyzed. A total of 31 putative protein biomarkers were identified—16 in plasma and 15 in muscle—with significant differences in abundance between the groups. These biomarkers were related to muscle structure, energy metabolism, heat shock proteins, oxidative stress, and proteolytic activity. Among the most prominent biomarkers were B2M, AHSG, APOA4, and HP-20 in plasma and PFKM, MYH2, PTER, GSTM1, and MYPN in muscle, which proved to be reliable predictors of textural quality. In addition, novel biomarkers have been identified in plasma, such as FETUB, SERPINA7, and AZGP1, which require further evaluation to determine their contribution to meat quality.

The meat proteome contains valuable information on unregulated processes or anomalies that occur during the manufacture of meat products. Meat classified as DFD (dark, firm, and dry) has been documented to have differential protein expressions compared to normal meat. In the native cattle breed Rubia Gallega, originating from Galicia, Spain, a significant change in fast skeletal myosin regulatory light chain two isoforms, characterized by a high level of phosphorylation, was observed. These isoforms showed a marked variation in DFD meat, being identified as biomarkers of this specific alteration in that breed [108].

Proteomics enables the detection of chemical and microbiological contaminants in food, ensuring the safety of meat and dairy products [109,110,111]. Abdelmegid et al. [112] used two-dimensional gel electrophoresis (2D-DIGE) coupled with LC-MS/MS to identify changes in the serum protein profiles of *S. aureus*-affected cows to identify biomarkers to facilitate disease diagnosis. They identified 28 highly abundant proteins in milk that were affected by *S. aureus*. Moreover, proteomic studies have contributed to advances in bacterial proteome mapping, the analysis of post-translational modifications, and the elucidation of pathogen-host interactions—key aspects in addressing antimicrobial resistance and discovering novel protein biomarkers [16].

In a different application, Nardiello et al. [113] investigated milk authenticity using a proteomic approach based on enzymatic digestion followed by mass spectrometry. The authors developed a method for detecting species-specific peptides in milk mixtures, allowing the identification of adulterants at levels below 1%. Milk samples were digested with trypsin and chymotrypsin and subsequently analyzed using LC-ESI-IT-MS/MS. Marker peptides specific to bovine milk, such as derivatives of β-lactoglobulin and αS1-casein, were identified and used to verify product authenticity.

Naveena et al. [114] developed an OFFGEL electrophoresis method together with a mass spectroscopy-based proteomic approach, which could be used in both raw and cooked meat mixtures containing beef, water buffalo, sheep, and goat meat. Another study addressed the challenge of authenticating ingredients in thermally processed meat products by protein degradation. Protein profiles were obtained from raw beef, chicken, duck, and pork, as well as from simulated adulterated samples of beef (chicken-res, duck-res, and pork-res) and their thermally processed versions using matrix-assisted laser desorption/ionization mass spectrometry with time-of-flight analysis (MALDI-TOF MS). Characteristic thermostable proteins were identified by analysis of the overlapping ion peaks between the raw samples and their processed counterparts using partial least squares discriminant analysis. Qualitative classification of thermostable proteins was achieved based on 36 characteristic thermostable proteins. In addition, quantitative analysis with partial least squares regression was performed to determine the percentage of adulteration in the simulated adulterated beef samples. The validity of the method was confirmed by a blind test, with an average precision of 97.4%. The limits of detection and quantification of the method were 5% and 8%, respectively, highlighting its practical utility for beef authentication [115].

There is growing concern regarding the allergen risk associated with the consumption of new protein sources [95]. MALDI-TOF mass spectrometry has been shown to be effective for the identification of cow milk proteins and their IgE-reactive isoforms in children. One study revealed that the most frequent allergies are αS1-casein (55% of patients), αS2-casein (90%), β-casein (15%), κ-casein (50%), β-lactoglobulin (45%), bovine serum albumin (45%), IgG heavy chain (95%), and lactoferrin (50%) [116]. Cow’s milk allergens can be divided into two main groups, caseins (αS1-casein, αS2-casein, β-casein, and κ-casein), which precipitate at pH 4.6 and 20 °C, and soluble serum proteins, such as β-lactoglobulin, α-lactoalbumin, lactoferrin, bovine serum albumin, and bovine immunoglobulins. However, the main milk allergens are casein, β-lactoglobulin, and α-lactoalbumin [117].

**Table 2:** Proteomic approaches in dairy and meat production systems.

Food Matriz	Techniques/Methods	Results	Application	References
Milk	nanoRP-UPLC-ESI-MS/MS; trypsin digestion; DIA and DDA acquisition	Identification of 132 modified peptides in 62 proteins (14 Age types). Increase in AGEs with processing severity, stable during storage. Formyl lysine was predominant	Quality Identification	[118]
	MALDI-TOF MS with reference spectra from >150 samples	Identification of animal species in feta and mozzarella cheeses. Proteomic modulation observed during mastitis.	Quality Identification	[119]
Meat	MALDI-TOF MS on *Longissimus thoracis* from heifers and steers	Validation of MALDI-TOF MS to differentiate cow, sheep, goat, and buffalo milk in cheeses	Quality Identification	[120]
	2D-PAGE, mass spectrometry, bioinformatics	Identification of *Pediococcus* and *Lactobacillus* strains capable of reducing β-lactoglobulin sensitization and hydrolyzing allergenic fragments	Composition Analysis	[121]
	OFFGEL electrophoresis (pH 4–7)	Four protein bands (Desmin, Pyruvate kinase, Myosin light/heavy chains) differentiated high vs. normal pH meats	Composition Analysis	[122]
	Trypsin/Lys-C digestion, LC-MS/MS (Q Exactive™ HF Orbitrap™)	Identification of 36 peaks in Uniprot database from meat exudates	Allergen Detection	[96]
	Shotgun proteomics of *Longissimus thoracis* in Arouquesa cattle	Proteins like HSP70 and laminin correlated with oxidative muscle stability	Authenticity Identification	[123]
	LC-MS for protein identification	Biomarkers such as VIM, FSCN1, SERPINH1, ALDH1A1, MYH4 identified as meat quality indicators	Quality Identification	[124]
	SDS-PAGE with image-based protein band quantification	Integration with OFFGEL electrophoresis and MS enabled high-resolution profiling of myofibrillar proteins	Quality Identification	[125]

Selection Criteria: Most recent scientific articles.

#### 3.1.3. Metabolomics

Metabolomics is an essential tool for gaining a detailed understanding of metabolite profiles in foods such as milk and meat. This approach allows for the evaluation of product freshness, quality, and authenticity by detecting molecular changes that occur during processing and storage [39]. This technology is essential for predicting the behavior of milk under different processing strategies [126]. The main applications of this technology in the food industry include optimizing food quality and safety, monitoring ripening processes, identifying metabolites associated with postharvest disorders, and exploring the connection between diet and health, which opens a promising field in nutritional metabolomics [127,128] (Table 3).

Metabolomics has become an indispensable tool in dairy science, as it enables the identification of biomarkers associated with animal health, productivity, authenticity, and the techno-functional properties of milk [129]. The characterization of the milk metabolome is a promising strategy for evaluating milk quality and authenticity [126], and it also facilitates the identification of bioactive metabolites with potential health benefits, such as milk allergens, thereby increasing the added value of dairy products [18]. For instance, Xu et al. [130] investigated the milk metabolome of cows experiencing negative energy balance and reported strong correlations between the animals’ energy status and specific milk metabolites, including glycine, choline, and carnitine. Using an untargeted metabolomics approach based on UHPLC-QTOF mass spectrometry combined with multivariate statistical analysis, researchers successfully discriminated against the chemical profiles of bulk milk used for hard cheese production from 103 dairy cows subjected to different feeding regimes, including corn silage, hay, and a mixed ration based on fresh forage and hay. This integrated approach underscored the discriminative value of both feed-derived compounds—such as phenolic metabolites likely originating from forage—and endogenous animal compounds like fatty acids, in determining milk quality [131].

Another study also analyzed the metabolic profiles of milk from healthy cows and cows with subclinical mastitis caused by Streptococcus agalactiae using time-of-flight gas chromatography mass spectrometry (GC-TOFMS). Data were processed using multivariate statistical analyses, such as principal component analysis (PCA) and partial least squares discriminant analysis (OPLS-DA), identifying 22 metabolites that were significantly different between the two groups. These include organic acids, carbohydrates, nucleotides, amino acids, and alcohols. The results showed that in cows affected by subclinical mastitis, metabolites such as phenyl pyruvic acid, uridine, glycerol, the homogentisic acid/4-hydroxyphenylpyruvic acid ratio, and the xanthine/guanine ratio was significantly elevated compared with those in healthy animals. These metabolic alterations were associated with changes in key pathways, such as carbohydrate metabolism (galactose, starch, and sucrose), the citric acid cycle (TCA), and arginine biosynthesis, highlighting the impact of mastitis on the metabolic profile of milk [132].

In addition, metabolomics facilitates food traceability, the evaluation of product mixture uniformity, and the detection of compounds in complex food matrices, thereby contributing substantially to quality control and the accurate prediction of product stability [133,134]. In this context, a study investigated the identification of metabolites capable of serving as early-stage indicators of cheddar cheese quality during ripening—an essential aspect of quality assurance in the cheese industry. Using nuclear magnetic resonance (NMR) spectroscopy, the aqueous extracts of cheddar cheese were analyzed over a 450-day ripening period. The ratios of citrulline and arginine relative to the total proton content in the aqueous phase emerged as key indices for assessing ripening progress, decreasing by 59% and 69%, respectively, over the maturation period. Furthermore, a lower-quality cheddar cheese batch exhibited higher concentrations of serine and β-galactose, lower levels of lactic acid, and a less mature sensory profile compared to a high-quality batch (Batch B). In contrast, metabolites such as tyrosine, tyramine, and lysine demonstrated strong correlations with the sensory attributes of mature cheddar cheeses, whereas β-galactose and glycerol were associated with those of less ripened cheeses [135].

Metabolomics also play a key role in food safety, enabling the detection of contaminants, the monitoring of veterinary drug residues, and the identification of potential adulterants in food, thereby helping to ensure compliance with international food regulations [136,137,138,139]. Rocchetti et al. [140] used an untargeted metabolomics approach based on ultra-high-performance liquid chromatography coupled with high-resolution mass spectrometry (UHPLC-HRMS) to analyze changes in the chemical profiles of cow’s milk with diets based on mycotoxin-contaminated maize silage. The results showed correlations between the quality of the contaminated silage (as part of the total diet) and the chemical composition of milk. A total of 628 significant metabolites were identified as being affected by the five levels of contamination, with amino acids and peptides being the most enriched classes (134 compounds). In addition, 78 metabolites with a projection significance score >1.2 were selected as the best discriminators of the predictive model. The most affected chemical classes were sphingolipids, together with purine- and pyrimidine-derived metabolites. Significant accumulation of oxidized glutathione was also observed in milk samples from silages contaminated with emerging *Aspergillus* toxins, suggesting a possible oxidative imbalance.

A prominent example of the use of metabolomics is its application to the reduction in methane emissions. In one study, a multi-platform metabolomic approach was used to analyze changes in milk metabolic profiles related to methanogenesis in dairy cows. Twenty-five primiparous Holstein cows at a similar stage of lactation, fed a uniform diet supplemented with a specific anti-methanogenic additive in the treated group (*n* = 12) or without the additive in the control group (n = 13), were included. Milk samples were analyzed using four complementary analytical methods: two non-targeted approaches (nuclear magnetic resonance and liquid chromatography coupled to time-of-flight and quadrupole mass spectrometry) and two targeted approaches (liquid chromatography coupled to tandem mass spectrometry and gas chromatography with a flame ionization detector). Data were filtered, selected, and normalized before analysis using a multivariate orthogonal partial least squares discriminant approach. All four methods were able to differentiate between the treated and control cows, identifying 38 discriminating metabolites affecting 10 metabolic pathways, including those related to methane metabolism. Among the metabolites highlighted, dimethyl sulfoxide, dimethyl sulfone, and citramalic acid were identified and associated with the rumen microbiome or co-metabolism between microbes and hosts, suggesting their possible relationship with methanogenesis. Furthermore, the additive reduced enteric methane production by 23% without affecting intake, milk production, or cow health [141].

On the other hand, in meat, this technology facilitates the identification of compounds related to flavor and texture, thereby improving quality control throughout the production chain. A recent study characterized the main metabolites in beef exudate to determine their relationship with color quality and oxidative stability of beef muscle. The metabolomic profile showed differentiation based on the aging period. Exudates from tenderloin muscles were found to have a higher concentration of oxidative enzymes, whereas exudates from loin muscles contained more glycolytic enzymes. In addition, higher concentrations of lipid metabolites, nucleotides, carnitine, and glycosides were found in loin muscle exudates and in those aged 23 days [125].

Another study investigated meat metabolomic profiles related to differences in quality attributes, specifically between high- and low-marbling beef groups, using nuclear magnetic resonance (NMR) spectroscopy. Significant differences in water and fat content were observed between the groups, with high marbled beef having higher concentrations of flavor-related compounds such as carnosine, creatine, glucose, and lactate. Using partial least squares discriminant analysis (PLS-DA), key metabolites such as N,N-dimethylglycine, carnosine, creatine, lactate, carnitine, sn-glycero-3-phosphocholine, betaine, glycine, glucose, alanine, tryptophan, methionine, taurine, and tyrosine, which are directly associated with marbling levels, were identified [142].

Furthermore, metabolomics provides detailed insights into metabolic changes induced by process modifications, nutrient intake, or the presence of underlying diseases, providing tools to design targeted interventions to optimize food quality and functionality [143]. In this context, Spears et al. [144] used non-targeted metabolomics by ultra-high performance liquid chromatography coupled to mass spectrometry (UPLC–MS) to analyze the metabolomic profiles of meat from conventional (CON) and organic grass-fed (GRA) systems. In addition, they evaluated postprandial metabolomic responses in human plasma after consumption of meat from both systems in a randomized crossover clinical trial with 10 healthy participants. The results showed that the feeding systems significantly influenced the metabolomic profiles of the meat and postprandial metabolic responses of consumers. In meat, differences in metabolites such as carnitines, fatty acids, and amino acids were identified, with a higher abundance of L-threonine in GRA meat and propionyl carnitine in CON meat. In contrast, modifications in metabolic pathways related to the metabolism of branched-chain amino acids (BCAA), fatty acids, and beta-alanine were observed in human plasma, showing a distinctive impact of each type of meat on postprandial metabolomics. These metabolites are linked to key biological processes such as oxidative stress, inflammation, and cardiovascular health.

**Table 3:** Metabolomic technologies in dairy and meat production system.

Food Matriz	Techniques/Methods	Results	Application	References
Milk	NMR-based metabolomics; different lactation stages in Friesian and native cows	Identification of 2355 chemical compounds, providing detailed chemical characterization	Composition Analysis	[145]
	LC-MS, ICP-MS, and NMR	Discriminate metabolites include lipids (fatty acids, phospholipids), amino acids, and plant-derived compounds.	Composition Analysis	[146]
	LC-MS/MS targeted metabolomics	296 metabolites identified in commercial bovine milk, with 1447 unique structures	Contaminant Detection	[147]
	Direct injection MS and LC-MS/MS for Aflatoxin M1 (AFM1) detection	AFM1 levels increased in milk from cows fed AFB1-contaminated diets	Contaminant Detection	[148]
	GC-FID and LC-MS for raw milk from healthy and subclinical ketosis cows	Increased tyrosine, leucine, carnitine, acetone in subclinical ketosis; reduced galactose-1-phosphate	Animal Health	[149]
	LC-MS/MS untargeted metabolomics	Decrease in creatinine, taurine, α-ketoglutarate in cows with subclinical ketosis	Composition Analysis	[150]
Meat	GC-MS and UHPLC-MS for beef and pork adulteration	Biomarkers such as leucine and creatine used for aging assessment	Adulteration Detection	[36]
	UPLC–MS/MS for beef muscle lipid profile	Correlation between degree of unsaturation in lipids and meat quality (unsaturated fatty acids, melting point)	Quality Identification	[151]
	NMR-based metabolomic profiling of beef (Nellore vs. Angus × Nellore)	Identification of 31 metabolites, including carnosine, betaine, and glycerol, correlating with sensory traits like flavor and tenderness	Composition Analysis	[152]
	UPLC-Orbitrap-MS and GC-MS for beef origin differentiation	24 metabolites identified as markers to differentiate beef origin (Australia, Japan, USA)	Origin Identification	[153]

Selection Criteria: Most recent scientific articles.

#### 3.1.4. Lipidomics

Lipidomics enables the precise control of lipid composition, which is essential for improving the fatty acid profile and meeting the demand for healthier products [17]. The lipid profile of milk is relevant not only for its nutritional quality but also for the investigation of beneficial compounds, such as omega-3 fatty acids [48] (Table 4). A recent study investigated differences in lipid profiles between bovine colostrum and mature milk using UHPLC-QTOF-MS lipidomics. A total of 335 lipids belonging to 13 subclasses were characterized, and 63 significantly differentially expressed lipids were identified. Of these, 21 lipids, including 5 phosphatidylethanolamines (PEs), 1 phosphatidylglycerol (PG), and 15 triacyl glycerides (TGs), showed significantly lower levels in mature milk than in colostrum. In contrast, the levels of 42 lipids increased in mature milk, including one cardiolipin (CL), nine diacylglycerides (DGs), nine dihexosylceramides (Hex2Cers), three hexosylceramides (HexCers), three phosphatidic acids (PAs), two phosphatidylcholines (PCs), 12 PEs, and three TGs. In addition, the correlations and metabolic pathways associated with these differential lipids were analyzed to explore the mechanisms responsible for lipid alterations during lactation. The seven most relevant metabolic pathways were the metabolism of glycerophospholipids, sphingolipids, and glycerolipids; the biosynthesis of glycosylphosphatidylinositol anchors; and the metabolism of linoleic, alpha-linolenic, and arachidonic acids [154].

Lipidomics has established itself as an effective tool to detect the adulteration of cow milk by the addition of plant lipids, a practice that, although aimed at increasing economic profits, compromises both the quality of dairy products and the health of consumers. England et al. [155] developed an innovative method based on matrix-assisted laser desorption/ionization mass spectrometry (MALDI-TOF MS) to discriminate between bovine milk and non-dairy milk, such as soy and coconut milk. This approach addresses the problem of food fraud by identifying unique lipid profiles for each type of milk, thereby enabling the detection of adulteration using specific markers. The analysis revealed that triacyl glycerides (TG) were predominant in coconut milk [TG (32:0), TG (34:0), TG (36:0), TG (38:0), and TG (40:0)], whereas phosphatidylcholines (PC) dominated in cow’s milk [PC (28:0), PC (30:0), PC (32:0), PC (34:1), and PC (36:1)], and soy milk showed higher concentrations of PC (34:1), PC (36:3), PC (37:3), and PC (39:5). Furthermore, the method proved to be able to accurately detect mixtures of adulterated milks, establishing clear relationships between adulteration ratios and variations in mass spectra.

Additionally, Ceciliani et al. [156] analyzed the milk lipidome in subclinical intra-mammary infections (IMI) caused by Coagulase-Negative Staphylococci using LC-QTOF-MS, comparing samples from 17 cows with NAS-IMI and 11 healthy cows. Their study identified 16 lipid subclasses and found that 597 lipids had significantly altered abundances, with notable changes in triacylglycerols and sphingomyelins, indicating inflammation in the bovine mammary gland. In parallel, the fatty acid profile in meat remains a critical parameter influencing quality attributes such as tenderness and flavor, enabling producers to adjust these traits to align with consumer preferences [157].

**Table 4:** Lipidomic tools in dairy and meat production systems.

Food Matriz	Techniques/Methods	Results	Application	References
Milk	LC-MS/MS analysis of phospholipids, sphingolipids, glycolipids, and ceramides	Identification of 514 lipid species across 15 classes	Composition analysis	[158]
	Infusion-electrospray mass spectrometry for triacylglycerides	Detection and quantification of over 100 TAG species in milk	Quality identification	[159]
	¹H-NMR and 1D TOCSY	Higher levels of α-linolenic acid, linoleic acid, and unsaturated fatty acids in organic milk; CLA isomers (9-cis, 11-trans) more abundant	Authenticity identification	[160]
	UPLC-Q-Exactive Orbitrap-MS	Soy milk: rich in phospholipids (PC, PE, PS, PG); Goat milk: high in MCTs, ω-3 and ω-6; Cow milk: intermediate lipid profile; 14 lipids identified as biomarkers	Adulteration detection	[161]
UHT Milk Reconstituted whole milk	UPLC-Q-Exactive Orbitrap-MS	Major lipid classes: PC (120 µmol/L), PE (150 µmol/L), SM (90 µmol/L)	Composition analysis	[162]
Meat	Intelligent surgical knife (iKnife) with REIMS	High precision lipid profiling (CV < 15% for most TAGs)	Quality Identification	[163]
	DART-QTOF (+) and LC-ESI-QTOF (+/−)	DART-QTOF: 852 peaks, 62 differential; LC-ESI-QTOF: 879 peaks, 165 differential; Clear clustering by country of origin (e.g., Brazil vs Canada)	Origin identification	[164]
	UHPLC-HRMS in positive and negative ion modes	Negative mode: detection of fatty acids, phospholipids, sphingolipids; Positive mode: phospholipids and glycerolipids	Authenticity identification	[165]

Selection Criteria: Most recent scientific articles.

### 3.2. Challenges and Opportunities

#### 3.2.1. Challenges

**Figure 1:**
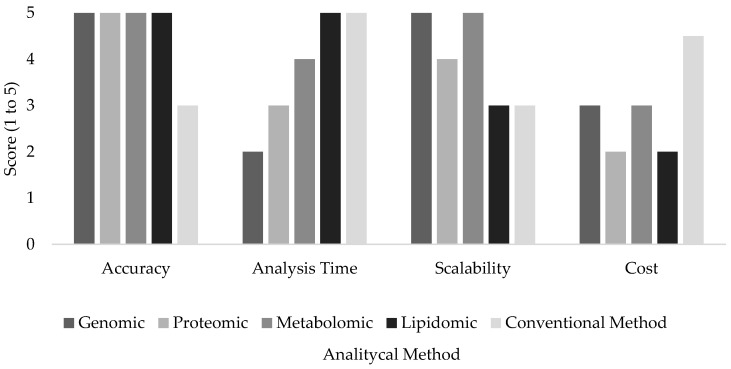
Comparison between omics’ technologies and conventional methods.

Omics technologies are increasingly employed in the agri-food sector owing to their high sensitivity, specificity, and analytical power. These technologies are applied to assess the quality, traceability, nutritional value, and safety of dairy and meat products. Compared to conventional analytical methods, omics approaches offer notable advantages, including enhanced qualitative discrimination and highly precise quantitative capabilities. Such strengths enable both targeted and untargeted detection of components, as well as their detailed molecular characterization—capabilities that are crucial for identifying food safety hazards, verifying product authenticity, and reinforcing quality control systems. Consequently, omics-based methods provide a more robust and comprehensive alternative to traditional techniques in food analysis [18,111]. Nevertheless, while conventional methods are generally faster and more cost-effective, their lower specificity and sensitivity limit their applicability in advanced research and in the analysis of complex food matrices (Figure 1).

The application of omics technologies within the dairy and meat industries presents considerable potential; however, it is also accompanied by substantial challenges. One of the principal obstacles lies in the complexity and volume of the data generated, which necessitates advanced analytical tools and specialized expertise for proper interpretation [166]. This complexity constrains the practical application of omics findings, particularly in contexts where resources and technical training are insufficient. Although omics technologies enable the identification of a wide variety of molecular changes, the biological interpretation of these results remains uncertain in many cases, thereby hindering the establishment of clear connections between molecular markers and desirable product traits, as well as food safety indicators.

Furthermore, the lack of standardized protocols for data preprocessing, such as peak alignment in metabolomics, complicates cross-study comparisons, thereby affecting the consistency and reliability of the results. This issue is further compounded by the scarcity of accurate phenotypic data, such as milk yield, meat tenderness, disease resistance (mastitis), or metabolic health indicators, which are often incomplete or fragmented. This limitation hampers the effective correlation of omics findings with practical applications aimed at improving product quality and safety.

Another critical challenge is the risk of false positives resulting from the high sensitivity of these technologies. For instance, the detection of mRNA from dead cells can lead to an overestimation of contamination risks, whereas false negatives may impede the identification of critical biomarkers related to product quality or safety. Moreover, the integration of multiple omics data layers, such as linking rumen microbiome profiles with meat quality traits, demands sophisticated bioinformatics processes that are often inaccessible to small- and medium-sized enterprises. Additionally, while technologies such as mass spectrometry and high-resolution sequencing are powerful analytical tools, they still face technical limitations, particularly in differentiating between viable and nonviable microorganisms during food safety assessments.

Economic and infrastructural barriers further restrict the widespread adoption of omics technologies within the sector. The high costs associated with acquiring specialized equipment, reagents, and skilled personnel constitute significant hurdles. Moreover, integrating omics data into existing food safety management systems and regulatory frameworks poses substantial challenges, as these systems are often not designed to accommodate the complexity and scale of information generated through omics approaches. This disconnect represents a major obstacle to the routine incorporation of omics technologies into quality and safety control strategies.

Furthermore, establishing statistically significant correlations between omics biomarkers and product quality traits, such as meat tenderness or flavor, as well as mastitis resistance genes and milk production traits, remains particularly difficult owing to the large biological variability between breeds and production systems. This variability complicates data interpretation and delays biomarker validation with practical utility. In addition, insufficient training in data analysis tools (Python) and statistical methodologies, especially in regions with limited technical infrastructure, further limits innovation. Simultaneously, the lack of regulatory frameworks adapted to omics-based data evaluation slows the approval and implementation of innovations, such as advanced pathogen detection technologies.

#### 3.2.2. Opportunities

Omics technologies offer transformative opportunities for the advancement of the dairy and meat industries. Through the integration of these tools, researchers are gaining deeper insight into the biological mechanisms underlying milk and meat production, thereby contributing to improvements in product quality, safety, and sustainability. These approaches facilitate the identification of genetic markers linked to desirable traits, such as higher milk yield, improved meat tenderness, and greater disease resistance, thus accelerating selective breeding programs and boosting overall productivity.

In the realm of food quality and safety, genomics, using techniques such as DNA metabarcoding, enables the detection of adulteration in meat products and pathogens such as Listeria spp. in dairy products. Metabolomics and lipidomics allow for the monitoring of biochemical changes during storage, ensuring the stability and safety of food products. From a genetic perspective, omics technologies facilitate the selection of animals with superior productive traits such as greater feed efficiency or enhanced disease resistance, thereby reducing production costs and greenhouse gas emissions.

**Figure 2:**
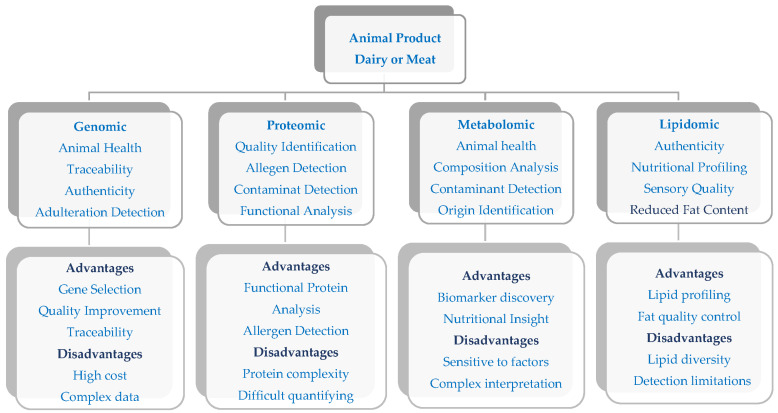
Omics application in dairy and meat products.

Moreover, omics technologies have driven the development of innovative products such as functional milk enriched with bioactive peptides or hybrid meats that combine animal and plant proteins to respond to new consumer trends. They also optimized animal nutrition strategies by studying the ruminal microbiome and promoting efficient and sustainable production systems. Furthermore, genomics strengthens food traceability and personalization, certifies product origins, and tailors food products to specific consumer needs (Figure 2).

### 3.3. Future Perspectives and Research Directions

Future studies in the field of milk and meat production should focus on the development of standardized and accessible platforms that facilitate the identification of robust biomarkers. These biomarkers are essential for improving the authentication, traceability, and nutritional profile of animal-derived products such as milk and meat. The adoption of these technologies should align with international standards, such as those promoted by the Food and Agriculture Organization of the United Nations (FAO), which advocates sustainable and safe practices in animal production, thereby ensuring global food security. Additionally, the implementation of specific standards issued by the International Organization for Standardization (ISO) such as ISO/TS 20428 [167] for genomics, ISO 14001 [168] for environmental management, and ISO 22000 [169] for food safety, will ensure the quality, reproducibility, environmental responsibility, and consumer safety of generated data and production processes.

From a sustainability perspective, omics technologies offer significant opportunities for optimizing industrial processes, minimizing the use of additives, and maximizing resource-use efficiency. Future research should prioritize the application of these technologies to enhance fermentation processes, extract functional ingredients, and reduce the environmental impact associated with animal production, thereby contributing to the consolidation of a more sustainable food system. In parallel, the detection of food fraud and product authentication remains a critical priority. It is imperative to develop faster, more sensitive, and more specific omics-based methods capable of proactively detecting contaminants and adulterants across the supply chain. The integration of artificial intelligence with omics platforms will enable early risk identification, strengthen process transparency, and foster greater consumer trust.

Nutritional personalization represents another promising area for future research. Omics technologies enable the development of foods tailored to the genetic and metabolic characteristics of individuals and populations. Research efforts should focus on deepening our understanding of the interactions among the genome, microbiome, and metabolism to create personalized functional products aimed at preventing chronic diseases and promoting public health.

To achieve a global impact, democratizing access to omics technologies is essential. Future studies must explore models for technology transfer, promote public–private partnerships, and design targeted training strategies to overcome economic and technical barriers, particularly in developing regions and among small and medium-sized enterprises. Standardizing protocols and harmonizing regulatory frameworks at the international level will also be crucial to ensuring effective and equitable implementation. Finally, the social acceptance of foods developed using omics technologies will largely depend on effective scientific communication. Future research should develop education and outreach strategies that clearly convey the benefits, safety, and traceability of these products, thereby promoting a culture of transparency, consumer confidence, and responsible innovation.

## 4. Materials and Methods

The revision was carried out through the scientific databases Scielo, PubMed, Google Scholar, Web of Science, and Science Direct, considering the literature published since the year 2000 and paying special attention to the last decade (2014–2025).

## 5. Conclusions

Omics technologies have significantly advanced the assessment of quality, safety, and authenticity in milk and meat production, offering a deeper and more accurate molecular understanding than traditional methods. Through the integration of omics technologies, it has become possible to identify robust biomarkers, detect adulteration, optimize nutritional profiles, and enhance traceability, thereby responding to growing consumer demand for safer, healthier, and more sustainable foods. Despite their great potential, challenges remain, particularly related to the high costs of implementation, the need for specialized equipment and personnel training, and the complexity of multi-omics data integration and interpretation. To fully leverage the transformative potential of omics technologies, future research should prioritize the development of standardized and accessible platforms that facilitate data integration and biomarker validation across diverse production systems. Additionally, advancing the application of omics in sustainable production processes, enhancing the detection of food fraud through artificial intelligence integration, and promoting nutritional personalization based on genetic and metabolic profiles are critical avenues for exploration. It is equally important to focus on democratizing access to these technologies, particularly in developing regions, through effective technology transfer models, public–private partnerships, and regulatory harmonization. Finally, although it is a technology that has been developed for more than 20 years, it has been difficult to integrate it into production systems.

## Data Availability

Not applicable.
